# Antioxidant and Antimicrobial Activity of Hydroethanolic Leaf Extracts from Six Mediterranean Olive Cultivars

**DOI:** 10.3390/antiox11091656

**Published:** 2022-08-26

**Authors:** Vida Šimat, Danijela Skroza, Giulia Tabanelli, Martina Čagalj, Federica Pasini, Ana María Gómez-Caravaca, Carmen Fernández-Fernández, Meta Sterniša, Sonja Smole Možina, Yesim Ozogul, Ivana Generalić Mekinić

**Affiliations:** 1 Department of Marine Studies, University of Split, R. Boškovića 37, HR-21000 Split, Croatia; 2 Department of Food Technology and Biotechnology, Faculty of Chemistry and Technology, University of Split, R. Boškovića 35, HR-21000 Split, Croatia; 3 Department of Agricultural and Food Sciences, University of Bologna, Viale Fanin 42, 40127 Bologna, Italy; 4 Department of Agricultural and Food Sciences, University of Bologna, Piazza Goidanich 60, 47521 Cesena, Italy; 5 Department of Analytical Chemistry, Faculty of Sciences, University of Granada, Avd. Fuentenueva s/n, 18071 Granada, Spain; 6 Department of Food Science and Technology, Biotechnical Faculty, University of Ljubljana, Jamnikarjeva 101, 1000 Ljubljana, Slovenia; 7 Department of Seafood Processing Technology, Faculty of Fisheries, Cukurova University, Adana 01330, Turkey

**Keywords:** olive leaves, antioxidant activity, antimicrobial activity, phenolic compounds

## Abstract

Phenolic profiles, antioxidant, and antimicrobial activities of hydroethanolic olive leaf extracts from six Mediterranean olive cultivars (Croatian: *Lastovka*, *Levantinka*, *Oblica*; Italian: *Moraiolo*, *Frantoio*, *Nostrana di Brisighella*) were investigated. As expected, various distributions of phenolic levels were observed for each cultivar and the total phenolic content showed high variability (ranging from 4 to 22 mg GAE/g of dry extract), with the highest amount of phenolics found in the *Oblica* sample, which also provided the highest antiradical (ORAC) and reducing activity (FRAP). The screening of individual compounds was performed by HPLC-PDA-ESI-QTOF-MS and the main detected compounds were oleuropein, hydroxytyrosol, oleoside/secologanoside, verbascoside, rutin, luteolin glucoside, hydroxyoleuropein, and ligstroside. While the antioxidant activity of the samples was relatively high, they showed no bactericidal and bacteriostatic activity against *E. coli* and *S.* Typhimurium; weak activity against *Staphylococcus aureus*, *Bacillus cereus*, and *Listeria innocua*; and inhibitory effects against *Campylobacter jejuni* at 0.5 mg dry extract/mL. The obtained results support the fact that olive leaf extracts, and especially those from the *Oblica* cultivar, could potentially be applied in various industries as natural preservatives and effective and inexpensive sources of valuable antioxidants.

## 1. Introduction

In Mediterranean countries, olive trees (*Olea europaea* L.) are commonly and traditionally cultivated to produce olive oil. Recently, the importance of the olive oil sector has increased worldwide due to the increasing consumption of oil for both its nutritional and health-related benefits. During the cultivation of an olive tree (pruning) and olive fruit processing, large quantities of agricultural residues remain unused. The main by-products are leaves, the amount of which depends on different factors, such as olive tree cultivar, environmental conditions, tree age, pruning practices, etc. [1,2,3,4], often leading to serious economic and environmental problems for producers. However, olive leaves could be converted into value-added products, rich in different phytochemicals and/or converted biofuels using chemical/biochemical methods [3]. This approach is valuable in increasing the profitability of olive cultivation, thus, enabling sustainable agricultural practices and a circular economy.

It is well known that olive leaves are a source of highly valuable and bioactive compounds with therapeutic and medicinal properties, which is why they have been used in traditional and folk medicine since ancient times. Due to their beneficial chemical composition, which comprises numerous classes of bioactive compounds of which phenolic substances are the most significant (such as oleuropein, verbascoside, rutin, tyrosol, and hydroxytyrosol), olive leaves also have the potential to be used as a source of antioxidants, antimicrobials, and natural preservatives, so can be applied in the food, pharmaceutical, and cosmetic sectors. The phenolic profile of olive leaves varies within cultivar and is influenced by the timing of sampling/harvest, climatic conditions, geographical origin, age and biological cycle of the tree, agricultural practices applied, and conditions during extraction/isolation, processing, storage, etc. [5,6,7,8,9,10,11,12,13,14].

While there are wide numbers of scientific studies on the phenolic profile and health benefits of olive oils, the amount of research dealing with composition and bioactivity of olive leaves is still scarce, especially on Croatian olive cultivars [7,15,16,17]. Further, many of the reported studies investigated the presence of only a few individual compounds, so there is a lack of comprehensive studies reporting full phenolic profile of the samples. Furthermore, recently, there is a great interest in an eco-friendly and green approach to the extraction of bioactives from olive leaves and different novel extraction technologies, which result in higher extraction yield, have shorter treatment time, apply less aggressive conditions (temperature, pressure, etc.), and are cost efficient in comparison to the conventional solvent extraction techniques that have been investigated [18,19,20,21,22,23,24,25,26,27,28,29,30]. 

Based on the above-mentioned literature, this study aimed to isolate and characterize bioactive constituents in olive leaves from six Mediterranean olive cultivars using ultrasound-assisted extraction (UAE) to gain a comprehensive insight into their chemical composition. For this purpose, liquid chromatography coupled with a photodiode array detector and electrospray ionization quadrupole time-of-flight mass spectrometer (HPLC-PDA-ESI-QTOF-MS) was used. Furthermore, to obtain information about the relation between the chemical composition and biological activity, the antioxidant and antimicrobial activity against foodborne pathogenic bacteria was also investigated by the multiple-method approach.

## 2. Materials and Methods

### 2.1. Plant Material and Extract Preparation

The samples investigated in this study were olive leaves of six olive varieties collected in October 2021 from standard orchards in Croatia (varieties *Lastovka*, *Levantinka*, *and Oblica* from the island of Pag and Marina) and Italy (varieties *Moraiolo*, *Frantoio*, *Nostrana di Brisighella* from Tuscany and Emilia Romagna), where these varieties are among the most popular. 

The fully expanded green leaves (samples of about 1 kg) were randomly collected manually from well-developed and healthy trees (5–10) of each variety (old between 20 and 35 years) from the middle part of olive shoots. The leaves were shade dried at room temperature for four to six days and ground (1 min in a high-speed grinder). The pulverized plant material was extracted with UAE (at 40 kHz) in triplicate for each sample using an ultrasonic bath (Transsonic Tp 310H, Elma Schmidbauer GmbH, Singen, Germany) using EtOH/H_2_O mixture as an extraction solvent (80:20, *v*/*v*) with sample:solvent ratio 1:100 g/mL at room temperature for 30 min. After extraction, the EtOH was evaporated and the extracts were freeze dried to extract the remaining water. The obtained dry extracts were used in further analyses.

### 2.2. HPLC-PDA-ESI-QTOF-MS Analysis of Phenolic Compounds

Qualitative and quantitative analyses of olive leaf extracts dissolved in 5 mL MeOH/H_2_O (50:50, *v*/*v*) were carried out using an ACQUITY Ultra Performance LC system equipped with a photodiode array detector with a binary solvent manager series with a Mass Quadrupole Time-of-Flight (QTOF) micro mass spectrometer (Waters Corporation, Milford, MA, USA) equipped with an electrospray ionization (ESI) source operating in the negative mode. The ESI source was operated with a capillary voltage of 2300 kV, cone gas flow of 40 L/h and desolvation gas flow of 11,000 L/h, source temperature of 100 °C and desolvation temperature of 500 °C, and scan range m/z 50–1500. An ACQUITY UPLC BEH Shield RP18 column (2.1 mm × 100 mm; Waters, Milford, MA, USA) with a particle size of 1.7 μm maintained at 40 °C was used for chromatographic separation which was obtained with a gradient program previously described by Čagalj et al. [31] using water + 1% acetic acid (*v*/*v*) and acetonitrile. The flow rate was 0.6 mL/min and injected sample volume was 2 μL. Pure standard solutions to obtain the calibration curves were used from the commercial-standard vanillin, ferulic acid, hydroxytyrosol, rutin, oleuropein, luteolin, luteolin 7-O-glucoside, verbascoside, apigenin, apigenin 7-O-glucoside, and pinoresinol that were purchased from Sigma-Aldrich (St. Louis, MO, USA). The compounds were monitored at 280 nm and MassLynx 4.1 software (Waters, Milford, MA, USA) was used to integrate and elaborate data [32].

### 2.3. Spectrophotometric Analysis of Total Phenolic Content (TPC) 

For the determination of TPC extracts were dissolved in EtOH (80:20, *v*/*v*) and antioxidant capacity. The total phenolic content (TPC) in the olive leaf extracts was determined by the Folin–Ciocalteu assay [33]. The absorbance was recorded by SPECORD 200 Plus (Edition 2010, Analytik Jena AG, Jena, Germany) and the TPCs of the samples were expressed as mg of gallic acid equivalents per gram of dry extract (mg GAE/g).

### 2.4. Antioxidant Capacity

The antioxidant activity of the samples was measured by three different antioxidant assays; 2,2-diphenyl-1-picrylhydrazyl (DPPH) radical scavenging activity and oxygen radical absorbance capacity (ORAC) methods which are based on the hydrogen atom transfer while the third method, ferric reducing antioxidant power (FRAP), is based on the electron transfer mechanism. The measurements were performed on a Tecan Microplate Reader, model Sunrise (Tecan Group Ltd., Männedorf, Switzerland) and Synergy HTX Multi-Mode Reader (BioTek Instruments, Inc., Winooski, VT, USA).

The FRAP method was measured according to the procedure reported by Skroza et al. [34]. The absorbance readings were taken after 4 min and the results were expressed as mM of Trolox equivalents (TE).

DPPH radical scavenging ability of the extracts was measured by the method described by Milat et al. [35] and the antioxidant activity was expressed as inhibition percentages of DPPH radical (% inhibition).

The inhibition of the action of free peroxyl radicals was monitored by the ORAC method according to the procedure reported by Čagalj et al. [31]. In this assay extracts were diluted 1000-fold prior analysis and results were expressed as µM of Trolox Equivalents (TE).

### 2.5. Antimicrobial Activity against Foodborne Pathogens and Spoilage Bacteria

Selected foodborne pathogens (Gram-positive *Staphylococcus aureus* ATCC 25923, *Listeria innocua* ŽM 39 and *Bacillus cereus* ŽMJ 164, and Gram-negative *Campylobacter jejuni* NCTC 11168) were included in the experiment. Microorganisms were obtained from the collection of microorganisms at the Laboratory for Food Microbiology at the Department of Food Science, Biotechnical Faculty, University of Ljubljana, Slovenia (designations ŽM and ŽMJ), the American Type Culture Collection (designation ATCC), and the National Collection of Type Cultures (designation NCTC). Tryptic soy agar and broth were used for revitalization and cultivation of bacteria and incubation under aerobic conditions at 37 °C for 24 h, except for *C. jejuni.* In this case, Karmali agar was used for revitalization and Mueller–Hinton agar and broth were used for further cultivation and incubation under microaerophilic conditions (5% O_2_, 10% CO_2_, 85% N_2_) at 42 °C for 24 h.

The antimicrobial activity of the extracts was evaluated by the broth microdilution method using 2-*p*-iodophenyl-3-*p*-nitrophenyl-5-phenyl tetrazolium chloride (INT) and resazurin as indicators [36]. Extracts were dissolved in dimethyl sulfoxide (32 mg/mL) and diluted before use. In a 96-well microtiter plate, 2-fold dilutions of the extracts were made with a final volume of 50 µL. The same volume of prepared inoculum (concentration of approximately 10^5^ CFU/mL) was added to each well and mixed. After incubation, 10 µL of INT or resazurin solution was added as indicators of bacterial metabolic activity. The minimal inhibitory concentration (MIC) was the lowest concentration at which no bacterial growth was detected as a reduction from colorless INT to red formazan or from blue resazurin to pink resorufin. The minimal bactericidal concentration (MBC) was the lowest concentration of the extract at which no bacterial growth was observed on agar plates after subcultivation of the bacterial suspension from the wells in which the MIC was determined and at higher concentrations.

### 2.6. Statistical Analyses

The statistical difference between the TPC and antioxidant activity of different cultivars was analyzed by analysis of variance (one-way ANOVA) followed by Fisher’s least significant difference procedure at a *p*-value of <0.05 using STATGRAPHICS^®^ Centurion XVI (StatPoint Technologies, Inc., The Plains, VA, USA).

## 3. Results and Discussion

### 3.1. Isolation and Chemical Characterization of Extracts

The recovery of phenolic compounds is a major challenge for the valorization of agro-industrial wastes and the selection of extraction methods is a crucial step in this process. Our previous study on olive leaves of known Croatian cultivars showed that alcohol–water mixtures are more suitable solvents for the extraction of phenolics, especially oleuropein [1], which was confirmed by the results obtained by Ghomari et al. [37] who reported that maceration with ethanol followed by water provided extracts with a high level of phenolics. Cör Andrejč et al. [14] investigated the effect of drying and extraction mode on oleuropein content in olive leaves and obtained the best results for air-dried samples when alcohol was used as an extraction solvent. Recently, novel and innovative technologies, including ultrasound-assisted extraction (UAE) [18,19,20], microwave-assisted extraction (MAE) [19,21,22], supercritical fluid extraction (SFE) [23,24], pulsed electric field extraction (PEF) [25], superheated liquid extraction (SHLE) [26,27], infrared-assisted extraction (IAE) [28], and high-voltage electrical discharge (HVED) [29,30], have been proposed to increase the recovery of phenolics from olive leaves, to shorten the extraction time, and prevent the degradation of the valuable phytochemicals. Regarding this, extraction of bioactive components in this research was achieved using safe and food-grade solvent (ethanol-water mixture) and their yield was additionally increased by the application of UAE as an eco-friendly and green technology.

The metabolites detected in the analyzed samples, belonging to different classes of flavonoids, simple phenols, secoiridoids, elenolic acid derivatives, and other phenolic compounds (in mg per g of dry leaf extract), are listed and summarized in Table 1. In total, 66 individual phenolic compounds were identified and the main compounds detected in the samples were oleuropein, hydroxytyrosol, oleoside/secologanoside, verbascoside, rutin, luteolin, ligstroside, and their derivates.

As expected, various distributions of phenolic levels were observed for each cultivar and the results were consistent with those previously reported [38,39,40,41,42] where the authors reported variations in phenolic levels among cultivars but also explained the obtained differences by environmental factors, such as harvest season and climatic conditions. The phenolic content in the olive leaf extracts showed high variability in a range of 4.2–22.3 mg/g, decreasing in the following order: *Oblica* > *Nostrana di Brisighella* > *Moraiolo* > *Frantoio* > *Lastovka* > *Levantinka*. In the study of Maletić Germek et al. [17], the highest phenolic level was detected in *Oblica* leaf extracts, among the other six Croatian cultivars.

Secoiridoids and flavonoids were dominant phenolic sub-groups in all samples. The samples of *Moraiolo*, *Frantoio*, *Nostrana di Brisighella*, and *Oblica* contained significantly higher amounts of secoiridoids, while in the extracts of *Lastovka* and *Levantinka,* the content of flavonoids (2.508 and 3.234 mg/g, respectively) was higher. Generally, *Oblica* leaves had the highest content of flavonoids (3.514 mg/g), secoiridoids (16.472 mg/g), and simple phenolics (0.707 mg/g). Furthermore, the content of elenolic acid derivatives in the *Oblica* sample (0.245 mg/g) was 1.6- to 10-fold higher than in the other samples.

Among the individual polyphenols, oleuropein was the major compound, accounting for 40–50% of the total phenolics. Although this share is relatively high, it is still significantly lower than levels reported by Medina et al. [40] for olive leaves, commercial leaf extracts, and infusions (74–94% of total phenolics) and by Maletić Germek et al. [17] for Croatian olive cultivars. In this work, the oleuropein level in *Oblica* was higher than in other cultivars, especially the oleuropein isomer c (10.217 mg/g). Previous studies showed that the concentrations of oleuropein in olive leaves vary considerably due to the type of cultivar, climatic and geographical conditions, sampling and drying techniques of the plant material, and the extraction parameters [7,13,17,41,42]. Irakli et al. [43] investigated the influence of UAE conditions (solvent type and concentration, extraction temperature, and time) on the extract yield of oleuropein and flavonoids from olive leaves and concluded that its yield increased significantly when the concentration of all solvents increased up to 50%. In addition, the harvest period can also affect phenolic content, but the reported results are contradictory. Romero et al. [44] reported the highest content of phenolics in the cold season, while Kabbash et al. [45] detected a significant increase in their concentration from autumn to spring. Furthermore, notable concentrations of hydroxyoleuropein isomers a and b and verbascoside isomers were found in the samples. Among other compounds, the *Oblica* sample contained considerable amounts of oleoside and hydroxytyrosol, simple phenolics naturally occurring in olives and olive oil. From the flavonoid group, mainly luteolin (luteolin glucoside isomers a and c) and apigenin derivatives (apigenin rutinoside isomers and apigenin glucoside) were found, while rutin was also detected in relatively high concentrations, 0.146 mg/g in *Lastovka* and 0.399 mg/g in the *Oblica* sample, which is in accordance with previously published results [16].

**Figure 1:**
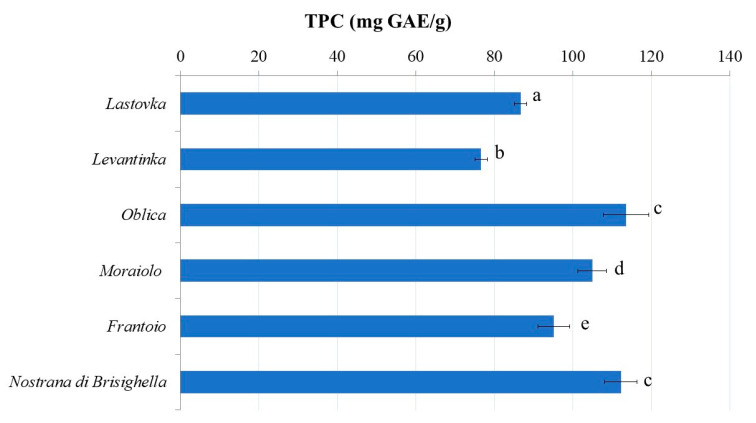
The content of total phenolics (TPCs) in olive leaf (in mg of gallic acid equivalents (GAEs) per g of dry extract (*n* = 4). Columns marked with different letters are statistically different (*p* < 0.05).

The analysis of TPC in the leaf extracts of the investigated cultivars (Figure 1), determined by the Folin–Ciocalteu (FC) method, also revealed variations (from 86.73 to 113.60 mg GAE/g), but the decreasing trend was the same, as confirmed by HPLC-PDA-ESI-QTOF-MS. *Oblica* and *Nostrana di Brisighella* showed the highest TPC, while the extract of *Levantinka* provided the lowest results. However, great differences obtained for the levels of phenolics from the chromatography technique in comparison to the similar levels of TPC using the FC method in *Oblica* and *Nostrana di Brisighella* are the result of low selectivity in the applied FC method, which is probably affected by other interfering substances presented in samples.

Debib and Boukhatem [46] studied the effects of solvents on olive leaf TPC and indicated that extraction solvent significantly affected the yield and profile of phenolic compounds, which was also confirmed in our preliminary study, while Hannachi et al. [19] reported MAE was a more efficient extraction method for polyphenolics, in comparison to the UAE. 

### 3.2. Antioxidant Activity

**Figure 2:**
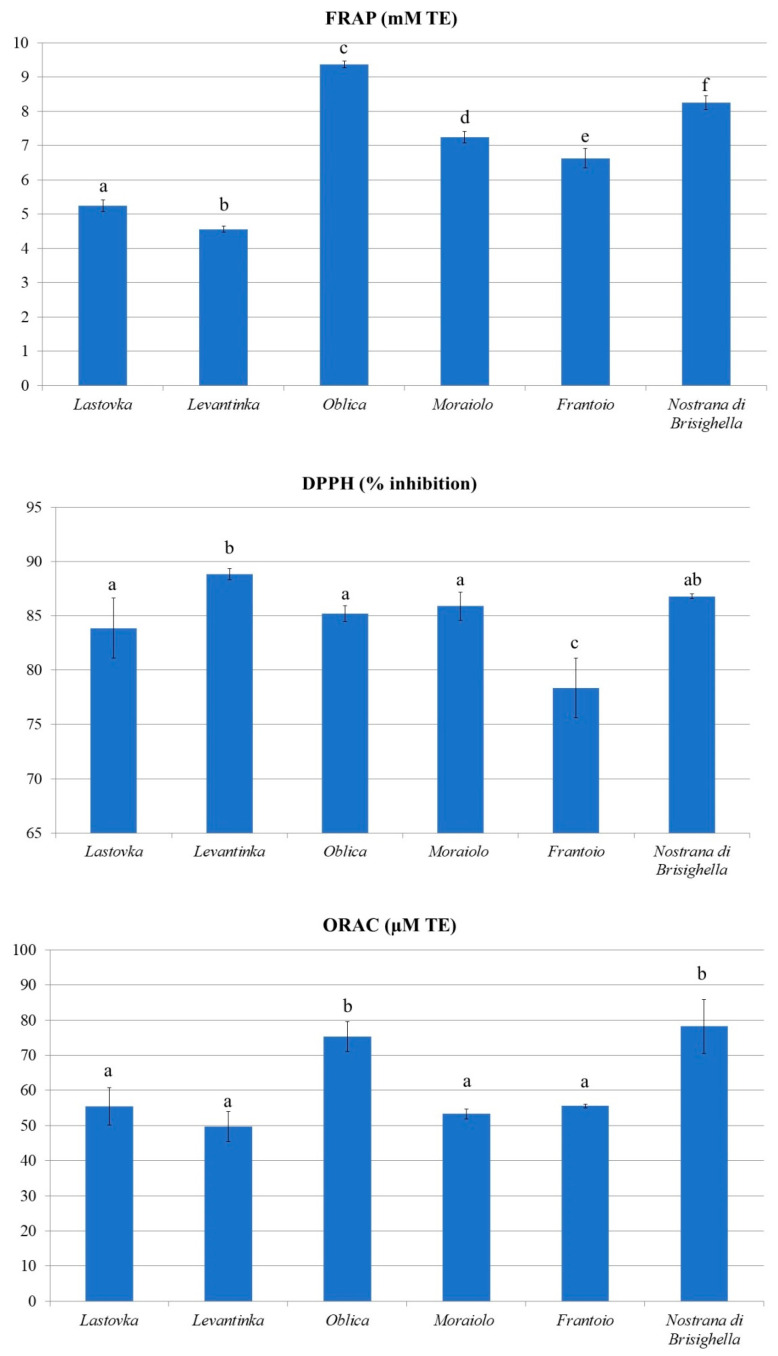
Antioxidant activity of olive leaf extracts detected by FRAP, DPPH, and ORAC method (*n* = 4). Columns marked with different letters are statistically different (*p* < 0.05).

The antioxidant activities of olive leaf extracts were investigated using FRAP, DPPH, and ORAC methods (Figure 2). A comparison of the obtained results with those previously published is very difficult, but in most cases, the general antioxidant activity is very high and usually explained by the rich phenolic profile of olive leaf extracts/samples [45,47,48] and chemical structure in the present phenolics (e.g., oleuropein, luteolin-7-O-glucoside acid, and hydroxytyrosol) [49].

In this study, the *Oblica* and *Nostrana di Brisighella* extracts with high TPC exhibited remarkable antioxidant activity detected by the FRAP method (9.36 and 8.25 mM TE, respectively), while the lowest reducing activity was observed for *Lastovka* (5.25 mM TE) and *Levantinka* (4.26 mM TE). The correlation between phenolic content and reducing activity detected by the FRAP assay has already been reported [50]. 

To determine the free radical scavenging capacity of different samples, the DPPH assay is considered a reliable and common method based on the scavenging ability of the sample antioxidants against stable free DPPH radicals [51]. The results for DPPH radical inhibition were similar and remarkably high (from 78.3 to 88.83% of inhibition) and did not correlate with TPC. 

Unlike other antioxidant activity assays, the ORAC assay relies on a common fluorescent probe, fluorescein, to measure the antioxidant activity of chain-breaking antioxidants against peroxyl radicals. According to the results of the ORAC assay, peroxyl radical scavenging ability was in the order: *Nostrana di Brisighella* > *Oblica* > *Frantoio* = *Lastovka*> *Moraiolo* > *Levantinka* (Figure 2) and was remarkably high (ranging from 49.71 to 78.19 µM TE). Moudache et al. [47] and Monteleone et al. [48] evaluated, in their study, the antioxidant activity of olive leaf extracts prepared by different solvents and the highest ORAC values were obtained in both studies for the aqueous ethanolic extracts.

### 3.3. Antibacterial Activity

In this study, the antimicrobial activity in the samples was evaluated against the main foodborne pathogens (Table 2). Concerning the microorganisms used, a sensitivity ranking for olive leaf extracts can be made in the following order: *C. jejuni* > *S. aureus* > *B. cereus* > *L. innocua* > *E. coli*, *S.* Typhimurium. 

Olive leaf extracts from the investigated cultivars did not show any antimicrobial activity against both *E. coli* and *S.* Typhimurium, while *C. jejuni* was the most sensitive bacterial target. All extracts provided the same MICs against this bacterial species (0.5 mg/mL) and MBCs (1.0 mg/mL), except for the value for the *Oblica* cultivar, which was, again, 0.5 mg/mL. The extracts of *Lastovka*, *Oblica*, and *Nostrana di Brisighella* showed stronger antimicrobial activity against *S. aureus,* with similar MIC and MBC values (2 mg/mL). Except for the *Levantinka* cultivar, the growth of *L. innocua* was not affected by the applied concentrations of the extracts. The bactericidal and bacteriostatic effect of all extracts on *B. cereus* was at a concentration of 8 mg/mL, except for *Lastovka*, which inhibited its growth at 4 mg/mL (both MIC and MBC).

Different studies have addressed the antimicrobial activity of olive leaf extracts against a wide class of microorganisms. Sudjana et al. [52] reported weak activity of olive leaf extracts against *C. jejuni*, *H. pylori*, and *S. aureus*, while Liu et al. [53] tested the antimicrobial activity of olive leaf extracts against *E. coli*, *Listeria monocytogenes*, and *Salmonella* Enteritidis and reported their inhibition at an extract concentration of 62.5 mg/mL. Martín-García et al. [54] detected antimicrobial activity of olive leaf extracts against *L. monocytogenes*, methicillin-resistant *S. aureus*, *E. coli*, *Salmonella* Typhimurium, and *Botrytis cinerea* (fungi) and reported MBCs ranging from 5.5 to 45 mg/mL, with the *Frantoio* cultivar extract being the most effective. Testa et al. [55] obtained MICs in a range of 2 and 5 mg/mL (*Gentile di Larino*) against spoilage bacteria, while the results reported by Hemeg et al. [56] for MICs and MBCs against *B. cereus*, *S. aureus*, *S.* Enteritidis, *E. coli*, and *Pasteurella multocida*, were 0.6–5 mg/mL and 0.6–2.5 mg/mL, respectively. Polyphenols form the extract, especially oleuropein, which is considered a natural antioxidant and was found to exhibit many other properties beneficial for human health, such as anti-inflammatory, anticancer, anti-obesity, antidiabetic, antihypertensive, cardio-, hepato-, and neuro-protective properties. The phenolic-rich olive leaf extracts could be used for the shelf-life prolongation of different foods (oils, meat, vegetables, baked goods, and dairy products) and preparation of functional food products. In addition, oleuropein, purified from *Domat*, *Edremit,* and *Trilye* cultivars, showed antioxidant and antimicrobial potential against *S. aureus*, *L. monocytogenes,* and *S.* Typhimurium [57]. Again, comparison of the results is extremely difficult due to the lack of uniformity in test methods.

## 4. Conclusions

This study aimed to gain substantial and thorough insight into the phenolic chemical composition of olive leaves from different varieties, which are usually unused by-products of olive tree cultivation and/or olive fruit processing. The results confirmed great variability between the results, both in phenolic profile and the concentrations of detected compounds, although in all samples, the dominant ones were oleuropein, hydroxytyrosol, oleo-side/secologanoside, verbascoside, rutin, luteolin, ligstroside, and their derivates. Especially interesting were the results for the *Oblica* sample (Croatian cultivar), which contained significantly higher amounts of key components and provided the highest antioxidant activity. The antimicrobial activity against tested Gram-positive bacterial strains failed or was weak, while the results obtained against *C. jejuni* showed notable inhibition (at 0.5 mg/mL). Therefore, olive leaf extracts can be considered an important and affordable natural source of antioxidants and antimicrobials that can find their application in various industries and the application of safe and food-grade solvents for the isolation of bioactive compounds opens new possibilities for their use.

## Figures and Tables

**Table 1:** Phenolic compounds in olive leaves determined by HPLC-PDA-ESI-QTOF-MS and expressed in mg analyte/g dry leaf extract.

		Quantification (mg/g Dry Leaf Extract)											
		*Lastovka*		*Levatinka*		*Oblica*		*Moraiolo*		*Frantoio*		*Nostrana di Brisighella*	
Compounds		x	SD	x	SD	x	SD	x	SD	x	SD	x	SD
1	Hydroxytyrosol-hexose	0.070	0.001	0.152	0.009	0.617	0.020	0.079	0.008	0.102	0.006	0.197	0.008
2	Oleoside	0.098	n.d.	<LOQ		0.387	0.012	0.104	n.d.	0.113	0.002	0.119	0.006
3	Hydroxytyrosol	0.009	0.002	0.021	0.005	0.090	0.004	0.123	n.d.	0.117	0.006	0.155	0.004
4	Oleoside/secologanoside	0.061	0.003	<LOQ		0.466	0.006	0.266	0.037	0.154	0.011	0.184	0.014
5	Gallocatechin	<LOQ		<LOQ		<LOQ		0.107	0.021	<LOQ		<LOQ	
6	Elenolic acid glucoside isomer a	<LOQ		<LOQ		0.067	0.004	0.003	n.d.	<LOQ		0.013	0.001
7	Elenolic acid glucoside isomer b	0.085	n.d.	0.024	0.006	0.097	0.001	0.100	0.007	0.082	0.001	0.086	n.d.
8	Elenolic acid glucoside isomer c	<LOQ		<LOQ		<LOQ		<LOQ *		<LOQ		<LOQ	
9	Oleuropein aglycon	0.093	0.002	<LOQ		0.093	0.004	0.010	0.014	0.004	0.006	0.085	0.006
10	Luteolin rutinoside isomer a	0.061	n.d.	0.096	0.003	0.090	n.d.	0.046	0.003	0.067	0.001	0.061	0.003
11	Luteolin-diglucoside isomer a	0.157	0.003	0.179	0.004	0.205	0.002	0.119	n.d.	0.106	0.001	0.173	0.012
12	Elenolic acid glucoside isomer d	<LOQ		<LOQ		0.081	0.003	0.049	n.d.	<LOQ		<LOQ	
13	Luteolin-diglucoside isomer b	0.036	n.d.	0.059	0.001	0.082	n.d.	0.020	0.001	0.019	n.d.	0.084	0.005
14	Demethyloleuropein	<LOQ		<LOD		0.037	0.005	0.081	0.002	0.284	0.024	0.046	0.007
15	Rutin	0.146	0.018	0.179	0.006	0.399	0.024	0.266	0.015	0.227	0.010	0.271	n.d.
16	Hydroxyoleuropein isomer a	0.208	0.002	0.089	0.016	0.037	0.001	0.313	0.011	0.171	0.003	0.250	0.003
17	Hydroxyoleuropein isomer b	0.203	0.007	0.088	0.013	0.033	0.002	0.333	0.010	0.178	0.004	0.248	0.002
18	Hydroxyoleuropein isomer c	<LOQ		<LOQ		<LOQ		<LOQ		<LOQ		<LOQ	
19	Luteolin rutinoside isomer b	0.083	0.001	0.097	0.001	0.079	0.001	0.073	n.d.	0.035	n.d.	0.086	0.005
20	Luteolin glucoside isomer a	0.424	0.002	0.473	0.016	0.557	0.003	0.459	0.015	0.478	0.008	0.464	0.016
21	Luteolin rutinoside isomer c	0.115	0.003	0.163	0.006	0.099	0.005	0.176	0.008	0.078	0.001	0.089	0.004
22	Hydroxyoleuropein isomer d	<LOQ		<LOQ		<LOQ		0.004	0.001	<LOQ		0.015	0.005
23	Verbascoside isomer a	<LOQ		<LOQ		0.860	0.047	0.562	0.018	0.595	0.053	0.970	0.022
24	Hydroxyoleuropein isomer e	<LOQ		<LOQ		<LOQ		0.010	0.004	<LOQ		0.004	0.005
25	Hydroxyoleuropein isomer f	<LOQ		<LOQ		<LOQ		<LOQ		0.025	0.003	<LOQ	
26	Luteolin glucoside isomer b	0.051	0.001	0.063	0.004	0.191	0.012	0.170	0.010	0.099	0.004	0.063	0.002
27	Oleuropein glucoside isomer a	<LOQ		<LOQ		<LOQ		<LOQ		<LOQ		<LOQ	
28	Apigenin rutinoside isomer a	0.167	n.d.	0.195	0.001	0.120	n.d.	0.096	0.002	0.113	0.001	0.127	0.006
29	Luteolin rutinoside isomer d	0.022	0.001	0.030	0.003	0.077	0.001	0.053	0.002	0.001	n.d.	0.091	0.004
30	Luteolin glucoside isomer c	0.409	0.001	0.459	0.013	0.507	0.006	0.478	0.016	0.530	0.001	0.461	0.001
31	Verbascoside isomer b	<LOD		<LOD		0.224	0.024	<LOD		0.004	0.001	0.008	0.002
32	Apigenin glucoside	0.219	0.007	0.323	0.015	0.257	0.003	0.140	0.008	0.222	n.d.	0.183	0.005
33	Oleuropein glucoside isomer b	<LOQ		<LOQ		<LOQ		<LOQ		<LOQ		<LOQ	
34	Oleuropein glucoside isomer c	<LOQ		<LOD		<LOQ		<LOQ		<LOQ		<LOQ	
35	Comselogoside	<LOQ		0.024	0.006	0.039	0.004	0.004	0.005	<LOQ		0.013	0.003
36	Verbascoside isomer c	<LOD		<LOD		0.251	0.017	0.087	0.016	0.120	0.010	0.297	0.041
37	Apigenin rutinoside isomer b	0.040	n.d.	0.054	0.001	0.014	0.001	0.047	0.005	0.039	0.001	0.012	0.001
38	Oleuropein glucoside isomer d	<LOQ		<LOQ		0.125	0.007	0.023	0.003	0.017	0.001	0.117	0.011
39	Oleuropein glucoside isomer e	0.018	0.001	0.022	0.002	0.125	0.002	0.024	0.002	0.008	0.001	0.056	0.007
40	Chrysoeriol-7-Oglucoside	0.117	0.005	0.244	0.019	0.342	0.002	0.189	0.008	0.329	0.007	0.238	0.008
41	Luteolin glucoside isomer d	0.137	0.001	0.240	0.011	0.375	0.003	0.121	0.008	0.152	0.002	0.280	0.015
42	Oleuropein glucoside isomer f	0.075	0.003	0.059	0.008	0.279	0.001	0.227	0.002	0.086	0.001	0.241	0.020
43	Oleuropein isomer a	<LOQ		<LOQ		0.131	0.002	<LOQ		<LOQ		<LOQ	
44	Hydro-oleuropein	<LOQ		<LOD		0.097	0.002	<LOQ		<LOQ		<LOQ	
45	Oleuropein isomer b	<LOQ		<LOQ		0.171	0.003	0.022	0.003	<LOQ		<LOQ	
46	2″-Methoxyoleuropein isomer a	0.042	0.001	0.018	0.009	<LOQ		0.174	0.019	0.113	0.002	0.007	0.002
47	2″-Methoxyoleuropein isomer b	0.040	n.d.	0.019	0.008	0.017	n.d.	0.187	0.020	0.122	0.002	0.020	0.002
48	Oleuropein glucoside isomer g	<LOQ		<LOQ		0.136	0.004	0.086	0.007	0.063	0.002	0.131	0.011
49	Oleuropein isomer c	0.414	0.037	0.325	0.055	10.217	0.148	3.162	0.082	2.928	0.017	4.146	0.106
50	Oleuropein isomer d	<LOQ		<LOQ		0.023	0.002	0.008	0.002	<LOQ		<LOQ	
51	Oleuropein isomer e	<LOQ		<LOQ		0.675	0.006	0.141	0.009	0.164	0.003	0.281	0.012
52	Luteolin	0.149	0.003	0.308	0.014	<LOQ		0.132	0.038	0.080	0.013	0.118	0.001
53	Oleuropein isomer f	0.037	0.008	0.021	0.012	2.456	0.042	0.460	0.023	0.546	0.007	0.798	0.060
54	Lucidumoside C isomer a	0.136	0.006	0.051	0.013	0.139	0.005	0.257	0.019	0.248	0.001	0.063	0.009
55	Lucidumoside C isomer b	0.139	0.009	0.046	0.012	0.127	n.d.	0.266	0.015	0.241	0.008	0.069	0.008
56	Ligstroside	0.047	0.008	0.019	0.007	0.639	0.012	0.156	0.010	0.152	0.007	0.283	0.005
57	Hydroxyoleuropein isomer g	<LOQ		<LOQ		<LOQ		0.047	0.001	<LOQ		<LOQ	
58	Lucidumoside C isomer c	0.001	0.002	<LOQ		0.010	0.001	0.027	0.002	0.041	0.002	<LOQ	
59	Oleuroside methyl ether	<LOQ		<LOQ		0.013	0.001	<LOQ		<LOQ		<LOQ	
60	Resinoside isomer a	0.054	0.003	0.005	n.d.	0.004	n.d.	0.012	n.d.	<LOQ		0.010	0.001
61	Oleuropein isomer g	0.002	0.001	<LOQ		<LOQ		0.093	n.d.	0.015	n.d.	<LOQ	
62	Oleuropein isomer h	0.008	0.002	<LOQ		<LOQ		0.096	0.001	0.020	0.001	<LOQ	
63	Oleuropein isomer i	0.009	0.003	<LOQ		<LOQ		0.072	0.005	0.008	0.001	<LOQ	
64	Oleuropein isomer j	0.007	n.d.	<LOQ		<LOQ		0.065	0.003	0.005	0.001	<LOQ	
65	Resinoside isomer b	0.082	0.007	0.041	n.d.	0.085	0.001	0.012	0.002	0.050	0.002	0.030	0.001
66	Resinoside isomer c	0.038	0.002	0.027	n.d.	0.030	n.d.	0.014	0.001	0.011	n.d.	0.019	0.001
Total	Simple Phenols	0.079	n.d.	0.173	0.014	0.707	0.024	0.202	0.008	0.219	n.d.	0.352	0.011
	Flavonoids	2.508	0.047	3.234	0.066	3.514	0.036	2.730	0.064	2.635	0.003	2.861	0.081
	Secoiridoids	1.638	0.079	0.779	0.148	16.472	0.242	6.720	0.258	5.705	0.023	7.177	0.271
	Elenolic acid derivatives	0.085	n.d.	0.024	0.006	0.245	0.006	0.152	0.011	0.082	0.001	0.099	0.001
	Other phenolic compounds	n.d.	n.d.	n.d.	n.d.	1.334	0.088	0.648	0.034	0.720	0.061	1.274	0.065
	Total phenols	4.310	0.127	4.209	0.233	22.273	0.395	10.443	0.162	9.361	0.082	11.764	0.428

* LOQ—limit of quantification; LOD—limit of detection; n.d.—not detected.

**Table 2:** Antimicrobial activity of olive leaf extracts expressed as minimal inhibitory concentration (MIC) and minimal bactericidal concentration (MBC) (in mg of dry extract/mL).

		Olive Varieties					
		*Lastovka*	*Levantinka*	*Oblica*	*Moraiolo*	*Frantoio*	*Nostrana di Brisighella*
*S. aureus* ATCC 25923	MIC *	2	4	2	4	4	2
	MBC	2	4	2	4	4	2
*L. innocua* ŽM 39	MIC	>8	8	>8	>4	>8	>8
	MBC	>8	8	>8	>4	>8	>8
*B. cereus* ŽMJ 164	MIC	4	8	8	8	8	8
	MBC	4	8	8	8	8	8
*E. coli* ATCC 11229	MIC	>8	>8	>8	>8	>8	>8
	MBC	>8	>8	>8	>8	>8	>8
*S*. Typhimurium ATCC 14028	MIC	>8	>8	>8	>8	>8	>8
	MBC	>8	>8	>8	>8	>8	>8
*C. jejuni* NCTC 11168	MIC	0.5	0.5	0.5	0.5	0.5	0.5
	MBC	1	1	0.5	1	1	1

* MIC—minimal inhibitory concentration; MBC—minimal bactericidal concentration.

## Data Availability

The data presented in this study are available in the article.
